# Effect of artesunate-mefloquine fixed-dose combination in malaria transmission in amazon basin communities

**DOI:** 10.1186/1475-2875-11-286

**Published:** 2012-08-20

**Authors:** Ana C Santelli, Isabela Ribeiro, André Daher, Marcos Boulos, Paola B Marchesini, Roseli La Corte dos Santos, Marize BF Lucena, Izanelda Magalhães, Antonio P Leon, Washington Junger, José LB Ladislau

**Affiliations:** 1 Programa Nacional de Controle da Malária, Secretaria de Vigilância em Saúde, Ministério da Saúde, Brazil; 2 Drugs for Neglected Diseases initiative, Rio de Janeiro, Brazil; 3 Instituto de Tecnologia em Fármacos - Farmanguinhos, Fundação Oswaldo Cruz, Rio de Janeiro, Brazil; 4 Universidade de São Paulo, São Paulo, Brazil; 5 Pan American Health Organization, Brasília, Brazil; 6 Universidade Federal de Sergipe, Sergipe, Brazil; 7 Secretaria Estadual de Saúde, Acre, Brazil; 8 Universidade do Estado do Rio de Janeiro – UERJ, Rio de Janeiro, Brazil

**Keywords:** Malaria, ACT, artesunate, mefloquine, Fixed-dose combination, P. falciparum, Brazil, Amazon

## Abstract

**Background:**

Studies in South-East Asia have suggested that early diagnosis and treatment with artesunate (AS) and mefloquine (MQ) combination therapy may reduce the transmission of *Plasmodium falciparum* malaria and the progression of MQ resistance.

**Methods:**

The effectiveness of a fixed-dose combination of AS and MQ (ASMQ) in reducing malaria transmission was tested in isolated communities of the Juruá valley in the Amazon region.

Priority municipalities within the Brazilian Legal Amazon area were selected according to pre-specified criteria. Routine national malaria control programmatic procedures were followed. Existing health structures were reinforced and health care workers were trained to treat with ASMQ all confirmed falciparum malaria cases that match inclusion criteria. A local pharmacovigilance structure was implemented. Incidence of malaria and hospitalizations were recorded two years before, during, and after the fixed-dose ASMQ intervention. In total, between July 2006 and December 2008, 23,845 patients received ASMQ. Two statistical modelling approaches were applied to monthly time series of *P. falciparum* malaria incidence rates, *P. falciparum/Plasmodium vivax* infection ratio, and malaria hospital admissions rates. All the time series ranged from January 2004 to December 2008, whilst the intervention period span from July 2006 to December 2008.

**Results:**

The ASMQ intervention had a highly significant impact on the mean level of each time series, adjusted for trend and season, of 0.34 (95%CI 0.20 – 0.58) for the P. *falciparum* malaria incidence rates, 0.67 (95%CI 0.50 – 0.89) for the *P. falciparum/P. vivax* infection ratio, and 0.53 (95%CI 0.41 – 0.69) for the hospital admission rates. There was also a significant change in the seasonal (or monthly) pattern of the time series before and after intervention, with the elimination of the malaria seasonal peak in the rainy months of the years following the introduction of ASMQ. No serious adverse events relating to the use of fixed-dose ASMQ were reported.

**Conclusions:**

In the remote region of the Juruá valley, the early detection of malaria by health care workers and treatment with fixed-dose ASMQ was feasible and efficacious, and significantly reduced the incidence and morbidity of *P. falciparum* malaria.

## Background

Artemisinin-based combination therapy (ACT) is considered the best available treatment for uncomplicated *Plasmodium falciparum* malaria, and is at the heart of the global strategy for malaria control [1]. One of the better documented form of ACT is that combining artesunate (AS) and mefloquine (MQ)(ASMQ) [2].

The use of ASMQ was considered in Asia as a strategy to mitigate resurgence of malaria and the intensifying spread of anti-malarial drug resistance well before the World Health Organization (WHO) recommended using ACT. Over the last 20 years, in the low transmission areas of the Thai Burmese border, the combination of AS and MQ has shown high efficacy and has been of great benefit in considerably reducing the transmission of multidrug resistant malaria, as well as reversing the trend of increasing MQ resistance. [2-8].

Safe, rapid, and reliably effective, the combination of AS and MQ is one of five forms of ACT currently recommended by the WHO as a first-line anti-malarial treatment. The WHO also recommends that fixed-dose combinations (FDC) be used whenever possible [1] to increase compliance to treatment. In 2002, in order to address the treatment needs of people most threatened by malaria and underscoring the need for public leadership, the Fixed-Dose Artesunate-Based Combination Therapies (FACT) Consortium, created by the Drugs for Neglected Diseases initiative (DNDi) and the Special Programme for Research and Training in Tropical Diseases (TDR), developed ASMQ as a (FDC). Within the FACT Consortium, Farmanguinhos was the first manufacturing partner of ASMQ FDC. By developing a FDC of well-established use, DNDi and its partners aimed to improve treatment compliance, extend its use in malaria endemic countries and fight more efficiently against resistance development.[9,10]. This user-friendly new tablet co-formulation, which simplifies treatment with a single daily dose of 1 or 2 tablets for three days, represents an innovation that could have considerable impact in the treatment of uncomplicated *P. falciparum* malaria. Fixed-dose combinations eliminate the possibility of patients taking only one component of the combination and are expected to improve patient compliance[1]. With specific presentations for children aged between 6 months and 11 years, ASMQ FDC addresses the needs of children, the primary victims of malaria worldwide.

**Figure 1:**
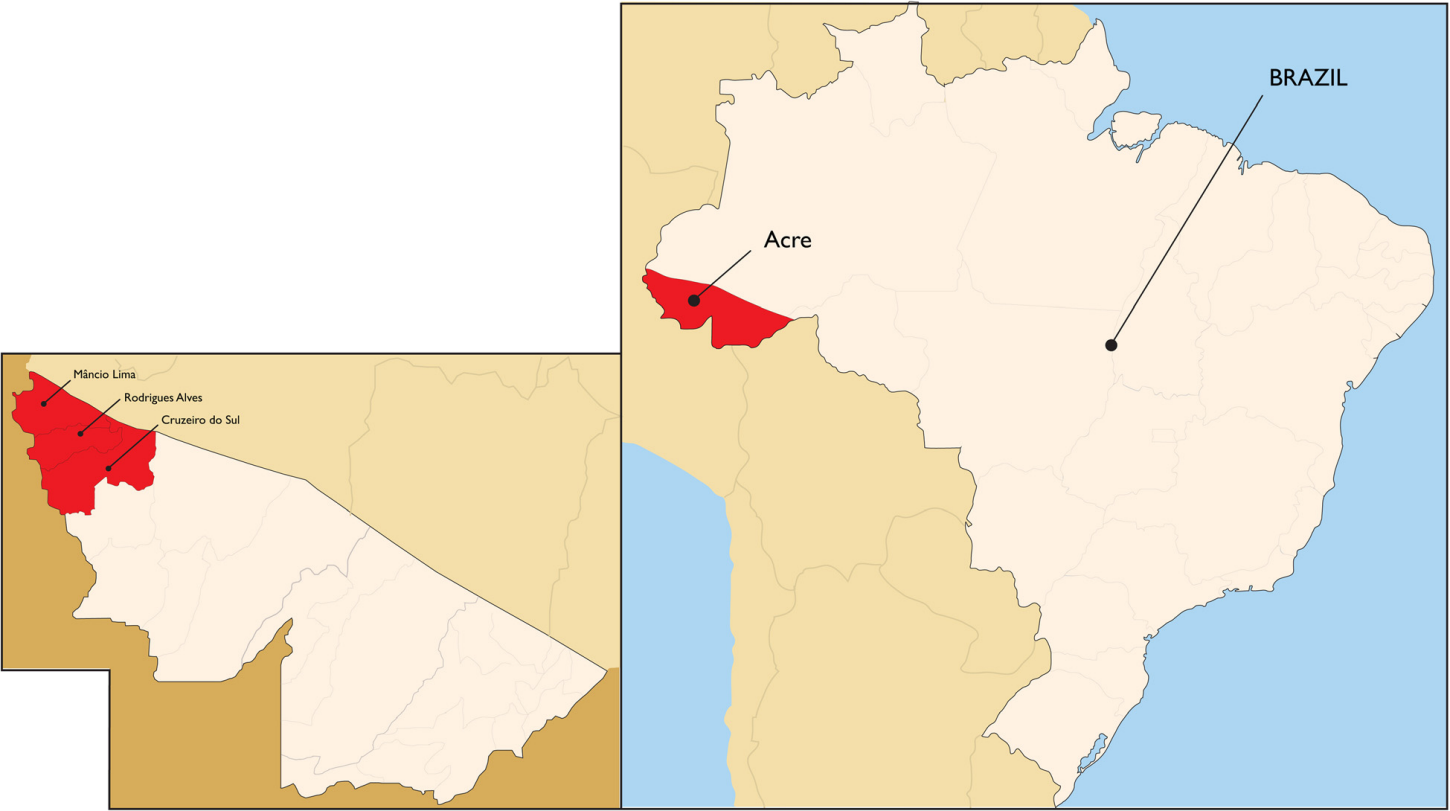
Location of the study area.

In 2008, approximately 320,000 cases of *P. falciparum* malaria were reported in Latin America, where Brazil has the highest malaria burden [11]. In Brazil, 99.8 % of malaria transmission occurs in eight states (Acre, Amapá, Amazonas, Mato Grosso, Pará, Rondônia, Roraima, Tocantins and part of Maranhão) that together form the Legal Amazon [12]. With a mean incidence of 500,000 cases/year, malaria affects all age groups equally, except individuals below one year and older than 60 years of age, for whom incidence is lower. Transmission increases during the seasonal peak, when climatic conditions are favourable to vector proliferation. New urban growth has been related to malaria burden in cities, such as Cruzeiro do Sul [12]. A decrease in malaria cases was observed as of 2006, which has been related to several factors, namely the introduction of ACT for *P. falciparum* malaria treatment, greater investments, capacity building on prevention and control, and epidemiologic data analyses, which allow a focus on malaria burdens in real time, both from decision makers and the populations involved [12]. Malaria risk in Brazil is classified according to the Annual Parasite Incidence (API). High risk areas have an API of ≥50/1000 inhabitants, intermediate areas of 10–49/1000 inhabitants, and low risk areas have an API <10/1000 inhabitants. From 2003–2007, in the Acre, Amazonas and Roraima states of the Legal Amazon, 79 municipalities were classified as high risk, including eleven with an API >300/1000 inhabitants, three of which were areas in the Juruá valley of the Acre State included in the present study: Rodrigues Alves, Mâncio Lima and Cruzeiro do Sul (Figure 1).

Efficacy studies initiated by the Amazon Network for the Surveillance of Antimalarial Drug Resistance (RAVREDA), created in 2001 to monitor *Plasmodium* resistance, showed cure rates below 90 % for quinine sulphate and doxycycline (at the time, the first-line regimen) for *P. falciparum* treatment, and prompted the introduction of ACT in 2006 by the National Malaria Control Programme (PNCM). Chloroquine and primaquine 0,50 mg base/kg during seven days remain the treatment for *P. vivax* malaria [13].

This article presents the results of a phase IV deployment study investigating the impact of fixed-dose ASMQ combination on *P. falciparum* incidence, *P. vivax* and *P. falciparum* ratio, and on the number of hospital admissions in three municipalities in the Amazon Basin (Brazil), characterized by high malaria incidence and concerns of increasing anti-malarial resistance to quinine-doxycycline. The study aimed to assess the suitability of replacing quinine sulphate and doxycycline as a national first-line treatment policy for children and adults with uncomplicated *P. falciparum* malaria in Latin America. This study is the largest to date on the programmatic use of ASMQ in Latin America.

### Study area description and population selection

The study included data collected between July 2004 and December 2008. The ASMQ intervention was carried out between July 2006 and December 2008 in three municipalities in the Juruá valley, within the state of Acre in Brazil: Cruzeiro do Sul, Mancio Lima and Rodrigues Alves (Figure 1). The municipalities were selected because they had recorded more than 20 *P. falciparum* malaria cases per month between 2003 and 2004, and had a stable population (defined by < 15 % proportion of imported cases), as well as supportive and cooperative local health authorities.

Acre state, situated in the most occidental region of Legal Amazon (Figure 1) has a population of 680,073 inhabitants; 15.2 % (103,371) of the population live in Cruzeiro do Sul, Mâncio Lima and Rodrigues Alves, and account for 86 % of Acre’s malaria cases. The state of Acre has a tropical climate, with temperatures ranging between 22–34 °C (72–93 °F), relative humidity of 60–85 %, and a rainy season that lasts from October through to April.

### Malaria epidemiology in Acre

Anti-malarial drug resistance has not been investigated in this region for almost two decades [14,15]. Acre borders Peru and Bolivia, where until recently first-line treatment regimens for *P. falciparum* malaria differed from those used in Brazil [13,16,17]. As a result, sustained population influx from these countries may affect the local patterns of drug-resistance [14,15].

Malaria incidence in some areas of the Amazon Basin rose between 2003 and 2005, which was attributed to several environmental and socio-economic factors, including climatic changes, new urban growth, cattle ranching and agricultural practices associated with deforestation) [16,18]. Public health strategies promoted by the PNCM were not fully implemented. The Ministry of Health initiated a collaborative task force of health managers in the Amazon region to coordinate population movements and to prioritize malaria surveillance, prevention and control[12,19].

In the Juruá Valley of Acre, Brazil, *P. vivax* predominates in urban populations, but in 2009, *P. falciparum* still accounted for more than half of the malaria burden in isolated river communities [16].

The most important malaria vector in Brazilian endemic areas is *Anopheles darlingi*, and the most vulnerable group consists of low-income workers (fishermen and gold miners) [20-22]. *Anopheles darlingi* is the main species captured outside and inside the homes of inhabitants of the Juruá valley, in a proportion of 4:1, respectively. The mean rate of *An. darlingi* per person/hour was 3.2 indoors and 11.5 outdoors. Both inside and outside feeding preferences are from ~8:00 p.m. until ~0:00 a.m. (Izanelda Magalhães, personal communication).

### Malaria and vector control programme

The control strategy of the Brazilian National Malaria Control Programme (Programa Nacional de Controle da Malária, PNCM) is based on early diagnosis and treatment, selective vector control interventions, early epidemic detection and involvement of local government and concerned populations. Malaria is diagnosed by rapid tests based on the immunochromatographic method or Giemsa-stained thick blood smears, according to PNCM training recommendations and quality control guidelines. Malaria treatment in Brazil is free of charge, as per government policy.

In 2000, malaria control programmes were decentralized to individual states of Brazil. In Acre state, as 90 % of total cases of malaria occur in the Juruá valley, a regional coordination body for malaria control was created. This organization includes support teams (diagnosis, epidemiology, vector control, health education and social mobilization), information systems as well as administrative and pharmaceutical assistance. In November 2005, intensified malaria control activities included active systematic screening of the population as routine practice, with weekly screenings performed by healthcare workers. Health surveillance workers in each locality collected thick blood smears, administered therapy and provided health education. These agents also prevented prescription mistakes and sought to improve treatment compliance. Local health facilities supported diagnosis and drug distribution. Drug supply was planned according to the number of cases reported for each type of malaria infection (*P. falciparum vs. P. vivax*). The Brazilian Ministry of Health regulated drug distribution and provided epidemiological data by mapping drugs received, distributed, and consumed.

By 2006–2007, the studied municipalities had a network of 73 diagnostic laboratories, 475 health surveillance workers, and 229 hospital beds. The private sector was also involved. Control strategy results were monitored by the Epidemiological Surveillance Information System Malaria module online database (SIVEP-malaria). Epidemiological and service indicators were evaluated on a weekly basis by the management team and all supervisors, ensuring that all information was disseminated widely.

Vector control strategies included outdoor thermo-nebulization and indoor chemical residual spraying with cipermetrine, and environmental management of larvae control until 2006. Biolarvicide was used in water tanks from December 2005 to July 2007. As of October 2006, the strategy focused mainly on vector control, with the goal of using indoor chemical spraying in 80 % of the highest risk area homes. In December 2007, an intervention with long lasting impregnated bed nets was initiated, where 7,000 insecticide-treated bed nets were distributed to cover 100 % of the population in 13 localities selected on the basis of epidemiological criteria: high disease burden, high proportion of *P. falciparum* infections, population age range, continuous access to treatment and diagnosis, good management of information systems, evidence of transmission inside the homes, and acceptability of bed nets to the population. These localities also had the least reduction in malaria incidence in previous years compared with other localities in the Juruá valley. Adequate anti-malarial drug control and supplies were available through the cooperation of health authorities and malaria control programs.

## Methods

The study was designed as an open-labelled, community-based intervention study in three municipalities of Acre state. These municipalities contained 73 localities, as notification units with laboratory and drug supply capabilities. The study included data collected routinely through the national surveillance system, SIVEP-malaria, between July 2004 and December 2008. The ASMQ intervention was initiated on July 2006, with completion on December 2008. The population within these municipalities was compared pre- and post-ASMQ intervention.

### Inclusion and exclusion criteria

All patients aged ≥6 months with an uncomplicated *P. falciparum* infection diagnosis, confirmed by thick smear enrolled in the public health system of the intervention municipalities were eligible for the study. The exclusion criteria were pregnancy and presence of clinical danger signs and other clinical features of severe malaria [23].

### Treatments and study procedures

There were no restrictions to the use of other medications, except for other anti-malarials and drugs with known interaction. Prior to study initiation, malaria health workers were trained in order to prevent uncontrolled use of other anti-malarials in the study areas. Special attention was paid to MQ single tablet therapy, which is no longer recommended and has been withdrawn from Acre state. Standard treatment with quinine, doxycycline or primaquine was available in case patients declined trial participation.

Participating subjects who met the inclusion criteria were treated with FDC ASMQ (manufactured by Farmaguinhos), according to age, using either a low dose (LD) tablet 25 + 50 mg (25 mg of artesunate and 50 mg of mefloquine base, as 55 mg mefloquine hydrochloride) or a high dose (HD) tablet: 100 + 200 mg (100 mg of artesunate and 200 mg of mefloquine base, as 220 mg of mefloquine hydrochloride).

Dosing was daily for 3 days and stratified by age: (i) 6–11 months of age: 1 ASMQ LD tablet, (ii) 1–6 years of age: 2 LD ASMQ tablets, (iii) 7–13 years of age: 1 HD tablet, and iv) 14 years of age and older: 2 HD tablets. Most of the time, treatment was directly observed, but for patients living in remote areas, only the first dose was directly observed and the remainder of the treatment was taken home. At study entry, diagnoses were performed by rapid test or thick smears, and patients were enrolled on the basis of a positive result. During the study period, rapid tests were performed in remote areas, but slides were collected to confirm the result by thick smear.

During the first six months of the study, samples of positive and negative malaria smears from every microscope technician were reread in blinded conditions for quality control, according to PNCM standard operational procedures. From November 2006, every positive only *P. falciparum* slide was revised, identified by a SIVEP number and filed under the month and notification area. As a routine procedure, 10 % of negative slides were re-examined. When conflicting results occurred, which was less than 1 % of cases, the microscopy technician was advised to attend a training programme.

The procedure to identify double entries and cure-verifying slides used the RecLink software duplicity routine [24,25] that generates a unique code for every individual. In order to improve software performance, records must be of good quality; therefore, the RecLink routine was alternated with manual verification procedures and repeated about 30 times. Every patient had the earliest positive slide defined as diagnosis date, and any slide between day 7 and day 40 after diagnosis was considered a follow-up slide.

### Safety evaluation

Adverse events monitoring was done by spontaneous reporting through the local pharmacovigilance system. In order to record these events, standard national regulatory agency forms were made available at the health facilities, diagnosis posts, and to every health surveillance agent.

### Ethical approval

The study was approved by the São Paulo University Ethics Committee. Participation in the study was entirely voluntary. Patients were informed of the risks and benefits relating to the study, and participants could withdraw from the study at any time. Written informed consent was obtained from all literate patients or guardians. Illiterate patients or guardians recorded their consent with a thumbprint. A witness confirmed both types of consent. Before starting the study, a state health team visited the communities involved to describe study objectives and methods. Communities and local healthcare providers also gave approval.

### Statistical analysis

In order to address the impact of the introduction of ASMQ in the Juruá valley region, three outcome variables were analysed: monthly incidence rates of P. *falciparum* malaria, the ratio between monthly incidences of P. *falciparum* and P. *vivax* malaria, and the monthly hospital admission rates due to malaria (ICD-10: B58 to B54). The period of analysis ranged from January 2004 to December 2008 (before and after introduction of FDC ASMQ).

Because the time series spanned five years, and the outcome variables were based on monthly incidence of the disease, the model had to take into account the effects of trend and seasonality.

The effect of ASMQ on the mean level of each outcome time series was evaluated in terms of main effects and interactions. Three main effects were evaluated, years, months and intervention, whilst the interaction involved intervention and months. The model, therefore, contemplated the time trend based on indicator variables for years and seasonality, with indicator variables for months, and the intervention impact with an indicator variable for the whole period after the introduction of ASMQ.

For each outcome variable two models were fitted: (a) only main effects of years, months and the intervention; and (b) the same main effects with the addition of an interaction between months and intervention. Hence, regarding the intervention, the first model exploits its effect adjusted for trend and seasonality, whereas the second model exploits its effect on if and how the intervention affected the seasonal pattern of the time series. The two models therefore evaluate different aspects of the intervention. The full model specification is presented in the expression below:

(1) $$\log\left(Y_{\mathrm{ij}}\right)=\beta_{0}+offset+\beta_{i}M_{i}+\gamma_{j}A_{j}+\alpha I_{\left\{ASMQ\right\}}+\delta_{i}M_{i}I_{\left\{ASMQ\right\}}$$

Where $\left\{Y_{\mathrm{ij}}:i=1,\ldots ,12;j=2004,\ldots ,2008\right\}$is the outcome time series,$\left\{M_{i}:i\neq 7\right\}$are month indicator variables, $\left\{A_{j}:j>2004\right\}$are year indicator variables, $I_{\left\{ASMQ\right\}}$ is the intervention indicator variable, *β*_0_ is a common intercept or the baseline (here July 2004), $\left\{\beta_{i}:i\neq 7\right\}$ are the monthly effects, $\left\{\gamma_{i}:i\neq 7\right\}$ are the yearly effects, *α* is the intervention effect, and $\left\{\delta_{i}:i\neq 7\right\}$ are the interaction effects between months and intervention. The offset was either the logarithm of the at risk population for rates, or the incidence of *P. vivax* malaria for the *P. falciparum/P. vivax* ratio.

The above is the expression for the second modelling approach. To obtain the expression for the first approach the interaction terms should be dropped from the formula.

As July 2006 was the first month after the start of the intervention, the month of July was chosen as the reference for the monthly pattern. 2004 was selected as the baseline year, as it represents pre-epidemic and pre-intervention levels of the time series. The intervention was implemented in June 2006, so the intervention period in the statistical analysis was regarded as beginning in July 2006 and ending in December 2008. The impact of the ASMQ intervention was assessed in comparison with the baseline, adjusting for the effects of other years and months. Thus, the coefficients of the remaining months and years represent the variation on the log-incidence rates in comparison to July 2004. The monthly counts were analysed and the model parameters estimated according to a quasi-Poisson estimation procedure and took into account an offset variable. Further, residual diagnostics were performed for each of the six adjusted models.

The two modelling approaches are presented as they address different questions;

- the main effect model establishes that the intervention had an effect on the mean level of the time series,

- whereas the main effect + interaction model establishes if the intervention had an effect on the monthly pattern (seasonality) of the time series, hence altering the mean level of the time series, albeit indirectly.

Data were analysed using the following software: Tableau 3.5, Microsoft Office Excel 2003, RecLink, and the R (R Development Core Team 2011) version 2.11.

## Results

### Description of population

The total population who received ASMQ between July 2006 and December 2008 amounted to 23,845 subjects in the Cruzeiro do Sul, Mancio Lima and Rodrigues Alves districts. The total population at risk in the three municipalities ranged from 96,496 to 103,799 between 2006 and 2008. The proportion of female participants was 43 %. There was no significant change in the total population of the different study municipalities in the Juruá valley during the observed period (January 2004 to December 2008). Over the intervention period, the yearly distribution of ASMQ-treated subjects living in the Juruá valley stratified by age showed that the majority (62.6 %) were aged 14 and over (Table 1).

**Table 1:** Yearly distribution of ASMQ-treated subjects in the Juruá valley stratified by age

	**<1 year**	**1 to 6 years**	**7 to 13 years**	**≥14 years**
**2006**	86 (0.7 %)	2,114 (17.4 %)	2,333 (19.2 %)	7,682 (62.7 %)
**2007**	72 (0.9 %)	1,303 (16.3 %)	1,569 (19.6 %)	5057 (63.2 %)
**2008**	38 (1.1 %)	667 (18.5 %)	698 (19.4 %)	2,195 (61.0 %)
**Total**	**196 (0.8** %**)**	**4,084 (17.2** %**)**	**4,600 (19.4** %**)**	**14,880 (62.6** %**)**

### *Plasmodium falciparum* incidence rates and efficacy evaluation

**Figure 2:**
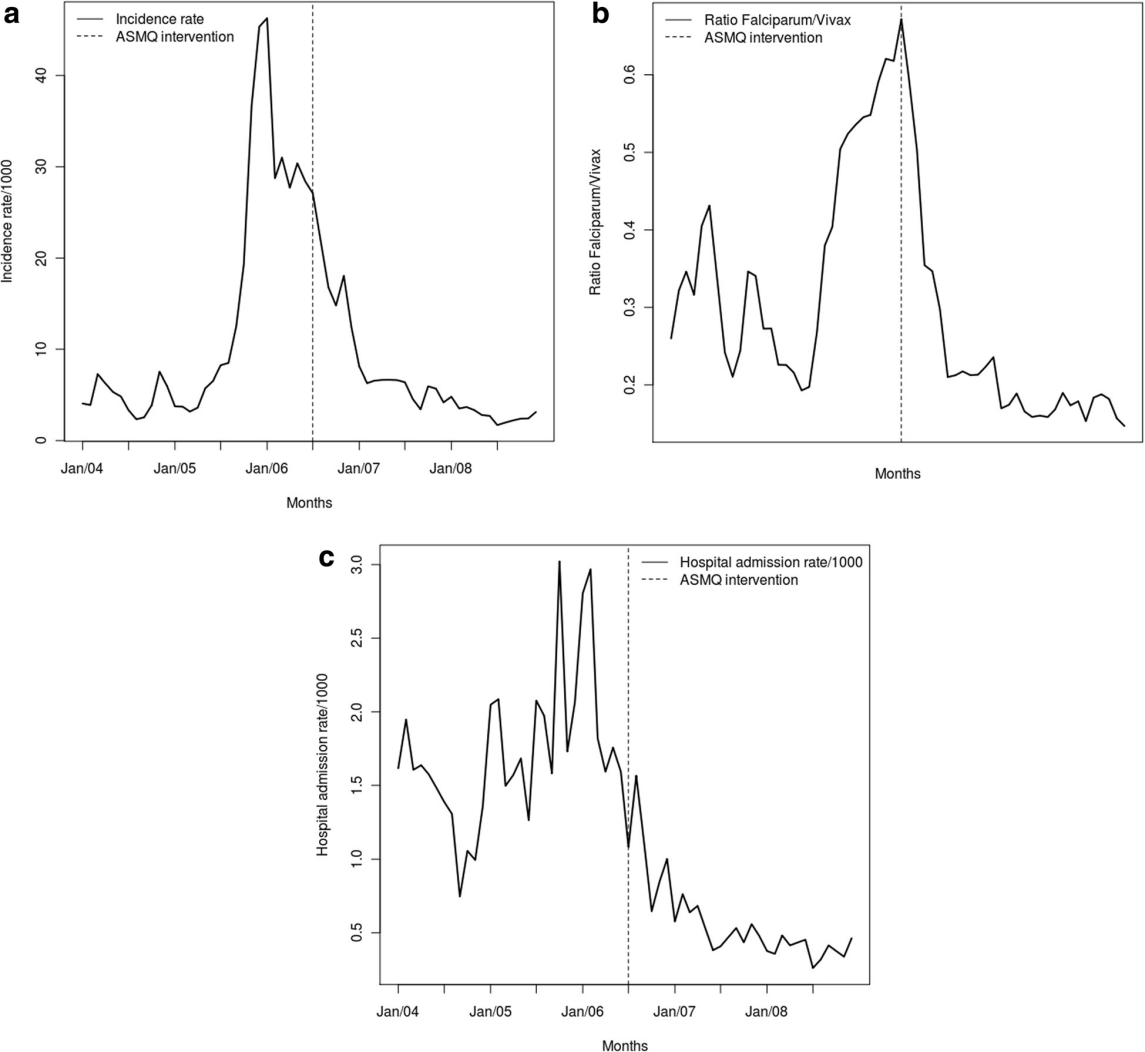
**Incidence rate of *P. falciparum *malaria (a), *P. falciparum/P. ****v ****ivax *ratio (b), and hospital admissions (c) due to malaria in the Juruá valley between 2004 and 2008.**

The *P. falciparum* malaria cumulative incidence rates (per 10,000 inhabitants) over one year were stratified by age range in the Juruá valley (Table 2). Following the introduction of ASMQ treatment in 2006, a large decrease in the *P. falciparum* yearly incidence was observed across all age groups of subjects living in the study area. The *P. falciparum* malaria incidence rate had sharply increased in the Juruá valley during the second half of 2005, with an epidemic peak at the beginning of 2006 before the introduction of ASMQ treatment (Figure 2a).

**Table 2:** ***Plasmodium falciparum *incidence rates (per 10,000 inhabitants) stratified by age (Juruá valley) **

**Year (population at risk)**	**<1 year**	**1 to 6 years**	**7 to 13 years**	**≥14 years**
**2004 (96496)**	5.58	56.43	55.56	60.22
**2005 (106882)**	22.64	148.58	161.81	164.33
**2006 (109827)**	67.95	298.59	327.63	307.84
**2007 (112755)**	22.88	68.15	79.64	71.44
**2008 (103799)**	13.38	38.58	38.01	33.61

Monthly incidence of *P. falciparum* malaria increased steadily over the first half of the study period, with a subsequent steady decline (Figure 2a). The peak *P. falciparum* malaria incidence occurred a few months before the introduction of FDC ASMQ therapy. The *P. falciparum/P. vivax* ratio increased in the same period up to the initiation of intervention, when there was a sharp drop (Figure 2b). The peak *P. falciparum/P. vivax* ratio coincided with the month of introduction of ASMQ. The pattern of malaria hospital admission rates represents a mix of the previous two series, however with a substantial reduction in the mean level at the end of the study period (Figure 2c).

Model parameter estimates transformed to the original scale are presented in Additional file 1, namely rate ratios in the case of incidence and hospital admissions, and ratios in the case of the *P. falciparum/P. vivax* ratio. Mutually adjusted increases (decreases) on the mean levels of the outcome are shown for a given month, year or intervention period. The different columns represent the adjusted model according to what was described in the methods section, thus six models altogether, two for each outcome. The two first columns correspond to incidence rates, the next two to the *P. falciparum*/*P. vivax* ratio, and the last two to hospital admission rates. Under each outcome, the first column shows results based on the first approach (main effects) whilst the second column presents results of the second approach (main effects and interactions). In order to address the difference between both approaches, the number of parameters fitted under each model and its quasi-deviance statistics are shown at the bottom of the table.

There was a significant decrease in the mean level of the time series for the three outcomes after the introduction of ASMQ, namely 0.34 (95%CI 0.20 – 0.58), 0.67 (95%CI 0.50 – 0.89), and 0.53 (95%CI 0.41 – 0.69) for incidence rates of *P. falciparum*, the *P. falciparum/P. vivax* ratio, and for malaria hospital admission rates, respectively. These results are adjusted for trend and seasonality (i.e. under the first modelling approach). It is important to notice the yearly effects pattern: for *P. falciparum* malaria incidence rates, 2005, 2006, and 2007 had significantly higher levels than 2004, whereas in 2008 there was no significant increase or decrease compared to 2004; for the *P. falciparum/P. vivax* ratio, 2006 had a significantly higher level than 2004; and for malaria hospital admission rates, 2005 and 2006 had significantly higher levels than 2004, but 2008 had significantly lower levels than 2004. In all outcomes, there was no statistically significant effect for months.

In addition, there was a marked change in the seasonal pattern of all three outcomes (i.e., under the second modelling approach). The incidence rates in the months of November (3.87, 95%CI 1.82 – 8.22) and December (4.50, 95%CI 2.14 – 9.46) before the intervention showed increases compared to July, whereas there were decreases of 0.74 (95%CI 0.42 – 1.32) for November and of 0.56 (95%CI 0.30 – 1.05) for December, after the intervention. A similar picture emerged for the *P. falciparum/P. vivax* ratio regarding the months of October, November, and December. It is important to notice that usually October marks the beginning of the rainy season in the Juruá valley region (Additional file 1).

**Figure 3:**
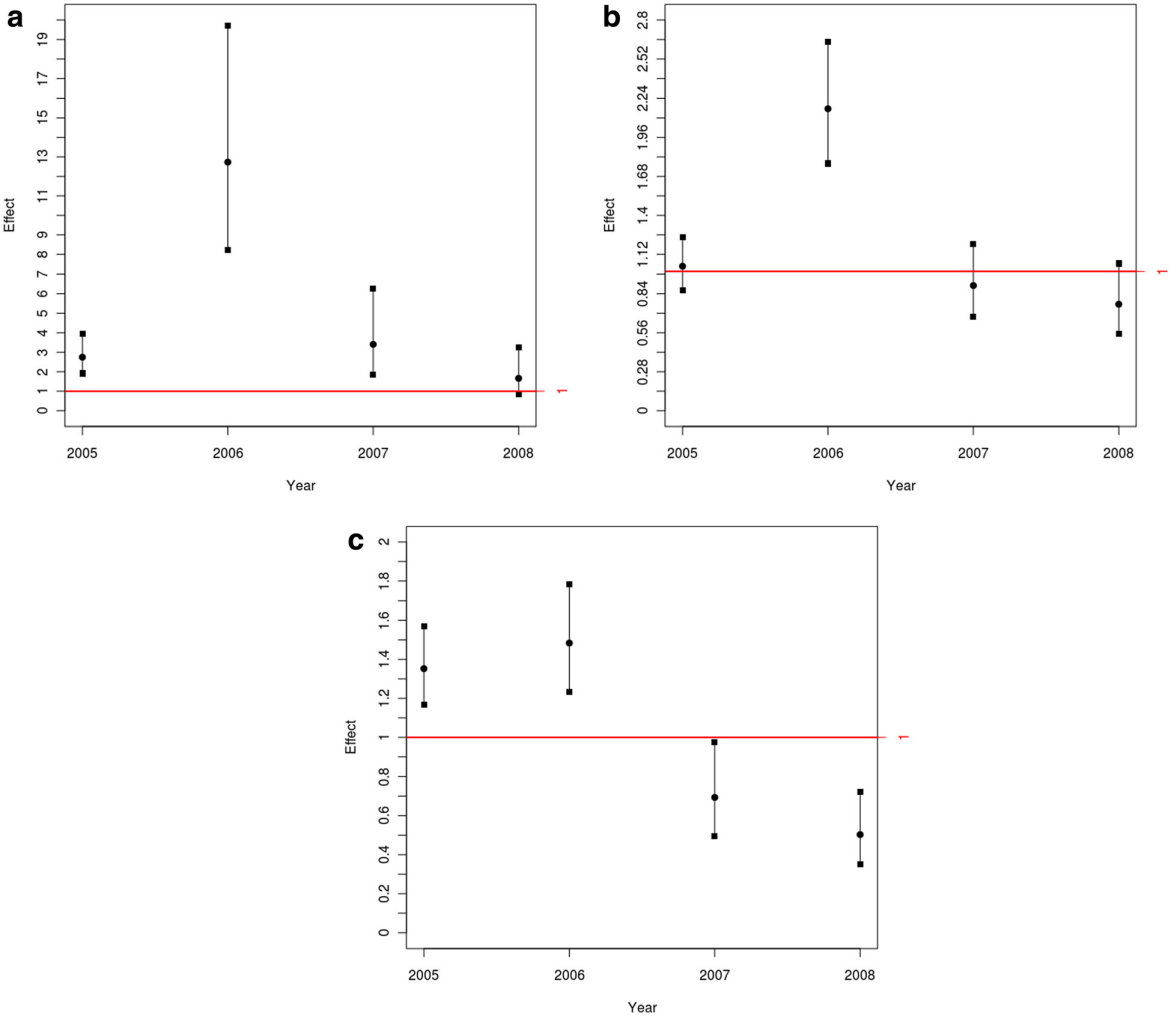
**Yearly effect under the interaction model for incidence rate of *P. falciparum *malaria (a), *P. falciparum/P. vivax *ratio (b), and malaria hospital admissions (c) in the Juruá valley between 2004 and 2008.**

Figure 3 shows the yearly effects for the three outcomes. Compared to 2004, the annual effect of the incidence rate of *P. falciparum* is statistically significant for every year except 2008, suggesting a return to pre-epidemic levels (Figure 3a). Regarding the *P. falciparum/P. vivax* ratio, Figure 3b shows that in 2007 the yearly effect of this outcome had returned to pre-epidemic levels. Finally, Figure 3c shows a marked and significant decrease in the yearly effect of malaria hospital admissions from 2007 onwards, suggesting that the intervention may have had a greater impact for this outcome.

**Figure 4:**
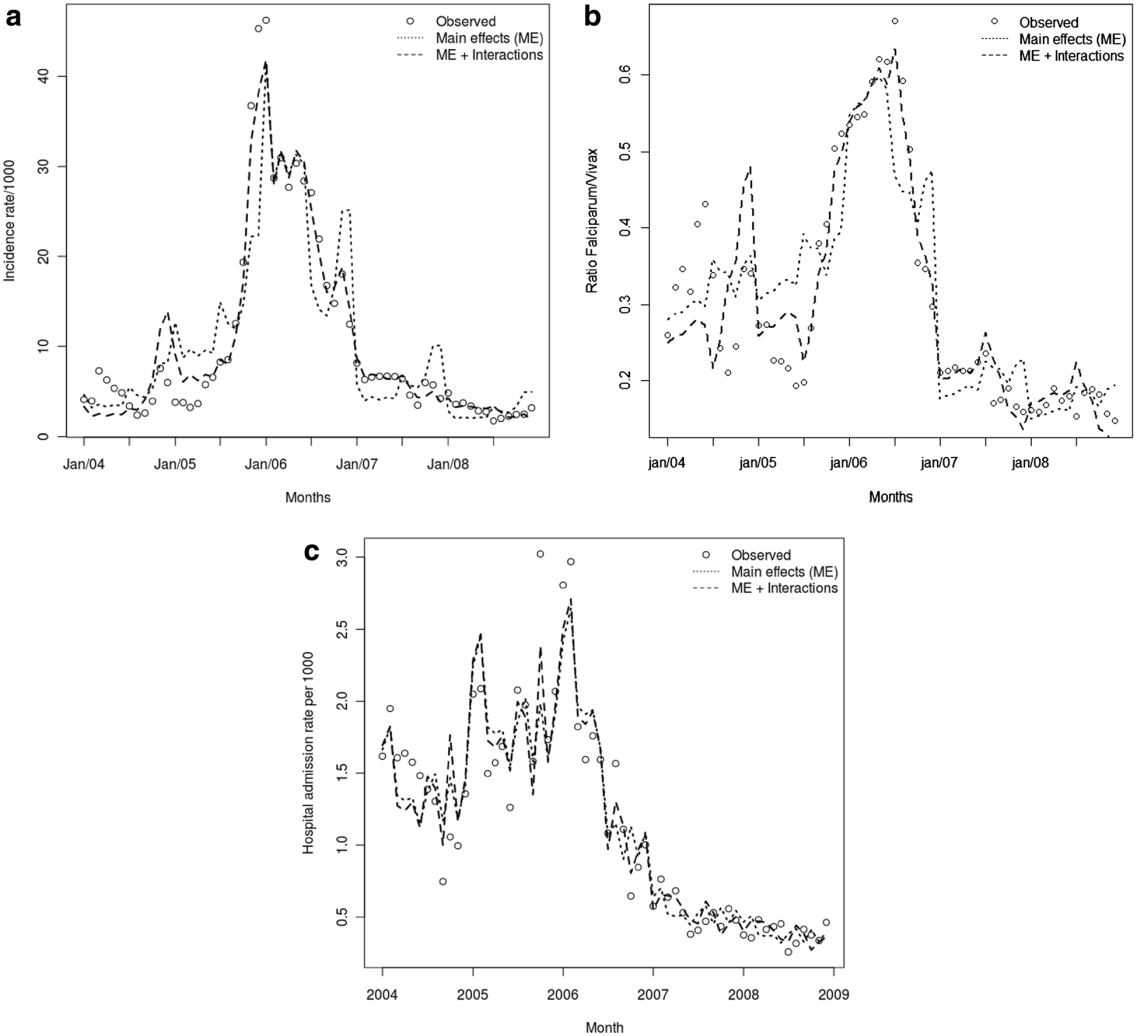
**Observed and predicted values under the main effects (ME) and the main effects and interactions models for incidence rate of *P. falciparum *malaria (a), *P. falciparum/P. vivax *ratio (b), and malaria hospital admissions (c) in the Juruá valley between 2004 and 2008. **(**a**).

Figure 4 shows the observed and predicted values for the three outcomes, under the different statistical approaches. Clearly under the second approach the models adjust more closely to the observed data, therefore justifying the inclusion of interaction between months and intervention.

### Safety evaluation

No serious adverse events, including deaths, relating to the use of fixed-dose ASMQ were reported. A single non-serious adverse report was recorded in 2007 in a seven-year old patient who experienced dizziness and vomiting, which are adverse events associated with MQ. Patient exposures were pooled from the SIVEP-malaria database. No direct adverse event reports were made to the toll-free pharmacovigilance telephone number for Farmanguinhos or to the national regulatory agency, ANVISA.

## Discussion

This intervention study is one of the largest successful trial implementations in a programmatic context in Latin America, and was performed in full collaboration with the Ministry of Health, both at the national and local levels, and the Pan-American Health Organization (PAHO). The study strengthened the local health management system in Acre state and showed that it is feasible to extend early malaria diagnosis and provide adequate treatment to remote communities. The positive effect of ASMQ was seen both in terms of malaria incidence and malaria-related hospital admission rates.

FDC ASMQ was assessed in the Juruá valley, an area with one of the highest incidences of *P. falciparum* malaria in Brazil. Indeed, prior to the intervention, the *P. falciparum* malaria incidence rate had increased dramatically during the second half of 2005, with an epidemic peak at the beginning of 2006 and the beginnings of a decline when ASMQ was introduced in June 2006. Various reasons were considered for the increase in *P. falciparum* malaria incidence in the Juruá valley during this period, including issues related to local malaria control management, vector control activities and decreased efficacy of the standard anti-malarial regimen (quinine and doxycycline).

When using 2004 as the baseline, pre-epidemic reference year, our results show *P. falciparum* malaria incidence rates returned to pre-epidemic levels by December 2008, preceded in 2007 by the *P. falciparum/P. vivax* ratio and hospital admission rates. ASMQ yielded a significant difference in the seasonal pattern of *P. falciparum* incidence rates, by eliminating the rainy season increase.

The *P. falciparum/P. vivax* ratio returned to pre-epidemic levels (2004) in the year immediately following the intervention (2007), before a fall in the cumulative incidence of *P. falciparum* cases, suggesting that the *P. falciparum*/*P. vivax* ratio may be more sensitive and specific than *P. falciparum* incidence alone as an epidemiological marker in areas of mixed transmission.

Data analyses were clouded because ASMQ was introduced in the declining phase of a steady malaria epidemic, after some 18 months, and in areas where vector control activities had been increased. Nevertheless, elimination of the seasonal peak and a significant decline in hospital admission rates are important and our data suggest ASMQ was the major contributor to these observations. A significant decline in severe malaria hospitalizations and malaria incidence were also documented in South Africa and on the Thai Burmese border following the deployment of artemether-lumefantrine and non-fixed ASMQ, respectively [6,26-28].

The inversion of the *P. falciparum/P. vivax* ratio is considered a key epidemiological indicator, as a signal of ACT activity and its potential impact on malaria mortality and morbidity. As the MQ dose is divided in three equal parts in the fixed-dose combination, it is expected that the fixed dose would have a better tolerability than non-fixed AS + MQ. A decrease in early vomiting with the new fixed-dose formulation has been previously reported [29]. Our study confirmed the good safety and tolerability profile of the combination. No serious or significant adverse events, such as convulsions or acute psychiatric emergencies were reported. One mild adverse event of dizziness and vomiting was transient, without the necessity for further intervention. The pharmacovigilance system described here was adapted to a rural setting, was simple, and relied on the passive detection of drug side effects, so lacked sensitivity, with consequential underreporting. The reporting of adverse events was emphasized during training of health workers and in informative leaflets. The low rate of reported side effects was attributed by the health workers to previous treatment with quinine, doxycycline and primaquine, whereby the population had grown accustomed to the frequent nausea and tinnitus associated with quinine. As the new fixed-dose ASMQ combination had better tolerability, it is thought that patients were less inclined to complain. Further data on safety in this area will be collected in the post-marketing setting (Farmanguinhos).

Studies have also shown that a fixed-dose ASMQ combination has improved bioavailability, compared to split-dose regimens [29], a beneficial pharmacokinetic effect for reducing the probability of developing *de novo* resistance. Moreover, the fixed-dose formulation may translate clinically into better compliance, thereby further contributing to limiting the development of drug resistance.

In areas with low levels of MQ resistance, initial recommendations by the WHO quoted lower doses of MQ in combination with an artemisinin drug, but based on pharmacokinetic and pharmacodynamic modelling data, it is now believed that MQ should be used at a dose of 25 mg/kg. Indeed, it was shown that initial use of a 15 mg/kg dose provided a greater opportunity for selection of resistant mutations and could thus lead more rapidly to resistance than using a dose of 25 mg/kg [30].

This phase IV deployment study had the intrinsic limitation of not being a randomized comparative study *versus* the current standard of care. However, our results show that FDC ASMQ represents an advance over the poorly tolerated and lengthy 7-day regimen of quinine and doxycycline.

## Conclusion

The fixed-dose ASMQ formulation was developed with the aim of addressing the treatment needs of those at risk of malaria, with ease of administration and a potential for improved treatment compliance. Based on convincing efficacy and safety data, this new product may have a considerable impact in the treatment of uncomplicated *P. falciparum* malaria in Latin America. The impact of this intervention was seen rapidly. The study strengthened the local health management system in the state of Acre and showed that extending early malaria diagnosis and providing adequate treatment to remote communities is feasible.

These results further support large-scale use of fixed-dose ASMQ and of ACT in general, a strategy that has shown similar results in other regions [26,27] and is recommended in all endemic regions to circumvent the spread of *P. falciparum* drug resistance and to reduce the impact of malaria [1].

Based on the data presented here, fixed-dose ASMQ was launched on a national scale. Ongoing investigations include continued pharmacovigilance monitoring, studies of ASMQ in pregnancy and an evaluation of ACT for the treatment of *P. vivax* malaria.

## Abbreviations

ACT: Artemisinin-based Combination Therapy; API: Annual Parasite Index; AS: Artesunate; ASMQ: Artesunate-Mefloquine; FDC: Fixed-dose combination; MQ: Mefloquine; *P*: *falciparum*; *Plasmodium*: *falciparum*; *P*: *vivax*; *Plasmodium*: *vivax*; PAHO: Pan-American Health Organisation; PNCM: Plano Nacional de Controle da Malária – National Malaria Control Programme; WHO: World Health Organization.

## Competing interests

The authors declare that they have no competing interests.

## Authors’ contributions

ACS coordinated the study and contributed to the writing of the manuscript; IR designed and coordinated the study and contributed to the writing of the manuscript; AD participated in coordinating and conducting the study and contributed to the writing of the manuscript, MB contributed to the study conception and design; PM participated in the study design and coordination and helped draft the manuscript; RLC participated in study design and coordination; MBFL conducted the study and helped draft the manuscript; IM conducted the study, and helped to draft the manuscript; APL conducted the statistical analysis and drafted the manuscript; WJ conducted the statistical analysis and drafted the manuscript, and discussed the study results; JLBL coordinated the study and helped draft the manuscript. All authors read and approved the final manuscript.

## Supplementary Material

Additional file 1 **Effect estimates and 95%CI for the indicator variables of years (trend), months (seasonality), and intervention (ASMQ) on three outcomes: *P. falciparum *Malaria Incidence Rates, *P. falciparum-P.vivax *Ratio, and Malaria Hospital Admission Rates in Vale do Juruá, Acre, Brazil, from January 2004 to December 2008.** Interaction effects between months and intervention are presented on every second column. Significant effects are highlighted. Click here for file
